# Association of geriatric nutritional risk index with all-cause hospital mortality among elderly patients in intensive care unit

**DOI:** 10.3389/fnut.2023.1117054

**Published:** 2023-03-23

**Authors:** Jiang-Chen Peng, Yi-Wei Zhu, Shun-Peng Xing, Wen Li, Yuan Gao, Wen-Wen Gong

**Affiliations:** ^1^Department of Critical Care, Ren Ji Hospital, School of Medicine, Shanghai Jiao Tong University, Shanghai, China; ^2^Department of Critical Care, Shanghai Baoshan Luodian Hospital, Shanghai, China

**Keywords:** geriatric nutritional risk index, malnutrition evaluation, hospital mortality, MIMIC-IV database, intensive care unit

## Abstract

**Background:**

Malnutrition is associated with poor outcomes for geriatric patients in intensive care unit (ICU). It is important to identify patients at risk of malnutrition and provide individual nutrition support. The assessment of malnutrition risk is not easy for these patients due to their cognitive impairment. Geriatric nutrition risk index (GNRI) is a simple and objective scoring tool to evaluate the risk of malnutrition in elderly patients. In this study, we aimed to see whether GNRI score was appropriate to predict clinical outcomes among geriatric patients in the setting of ICU.

**Materials and methods:**

Elderly patients with age ≥ 65 years were extracted from Medical Information Mart for Intensive Care IV (MIMIC-IV) database. Categories based on GNRI were classified as major risk (GNRI <82), moderate risk (GNRI 82 to <92), low risk (GNRI 92 to ≤98), and no risk (GNRI >98). The primary outcome was all-cause hospital mortality. Multivariable Cox proportional hazards regression models and restricted cubic spline were used to investigate associations of GNRI with hospital mortality, respectively. A two-piecewise linear regression model was applied to examine the inflection point of GNRI on hospital mortality. To reduce selection bias, propensity score matching (PSM) was used in a 1:1 ratio.

**Results:**

A total of 3,696 geriatric patients were finally included with median age 75 (69, 81) years. The prevalence of major risk was 28.6%. In the fully adjusted model, GNRI categories featured a negative trend with hospital mortality (*p* for trend = 0.037). Restricted cubic spline analysis demonstrated an L-shaped relationship between GNRI and hospital mortality before and after matching. The inflection point was 78.7. At the left side of inflection point, GNRI levels were significantly negatively associated with hospital mortality (HR = 0.96, 95% CI: 0.94–0.98; *p* < 0.001) and featured no significant relations at the right side. Multiple linear regression also showed that GNRI was negatively associated with length of stay in hospital.

**Conclusion:**

The major risk of malnutrition defined by GNRI was able to predict poor prognosis for geriatric patients admitted to ICU.

## 1. Introduction

According to the population division of United Nation, the proportion of persons aged 65 or over is projected to increase globally between 2022 and 2050. The older population is estimated to reach 994 million by 2030 and 1.6 billion by 2050 (1). Malnutrition appears to be a common issue among older population with the aging process, ranging from 10 to 50% due to different diagnostic criteria (2). For hospitalized older patients, only 14% of them are nutritional well-being according to a multinational retrospective pooled analysis (3). For the critically ill geriatric patients in the intensive care unit (ICU), stress-related catabolism and proinflammatory cytokines might further result in deterioration of nutritional status after admission to ICU (4), which leads to prolonged length of stay, increased incidence of infection and poor prognosis (5). Therefore, it is important to identify elderly patients with malnutrition risk in a timely manner and treat them adequately so as to minimize the development of malnutrition and reduce its deleterious results.

However, dozens of nutrition screening tools have been proposed and there is no tool to be currently considered the gold standard for screening risk of malnutrition (6). Mini Nutritional Assessment (MNA) is recommended by the European Society for Clinical Nutrition and Metabolism (ESPEN) (7). While, Nutritional Risk Screening-2002 (NRS-2002) and Nutrition Risk in the Critically ill (NUTRIC) score are suggested by The American Society for Parenteral and Enteral Nutrition (ASPEN) (8). However, these tools have limitations for clinical application. First, these assessments require a series of questionnaires, which are too complex to be suitable for older patients with difficulties in communication and cooperation. Besides, Acute Physiology and Chronic Health Evaluation II (APACHEII) score are necessary for NRS-2002 and NUTRIC score, which impedes screening due to spending a lot of time and effort (9). So, it is necessary to find a rapid, simple and objective tool that allows clinicians to screen for malnutrition risk among older individuals admitted to ICU.

Geriatric nutritional risk index (GNRI) was developed by Bouillanne et al. in 2005 and was designed specifically to assess nutritional status of the aging population (10). The calculation of GNRI is based on serum albumin level and body mass index (BMI). Several studies have validated that low GNRI score was associated with poor prognosis in patients with heart failure (11), acute coronary syndrome (12), chronic hemodialysis (13), malignancy (14), and acute ischemic stroke (15). However, the association between GNRI and prognosis in ICU is limited. In this study, we aimed to investigate whether GNRI score was able to predict clinical outcomes among geriatric patients in the setting of ICU.

## 2. Materials and methods

### 2.1. Data source

We conducted this retrospective study based on Medical Information Mart for Intensive Care IV version 1.0 (MIMIC-IV v1.0) database. MIMIC-IV, a large and public database, contains comprehensive data of more than 60,000 patients admitted to the ICU at Beth Israel Deaconess Medical Center from 2008 to 2019 (16). One author (P.J.C) has completed the online training course of the National Institutes of Health and obtained access to the database (record ID: 41046393). The project was approved by the institutional review boards of the Massachusetts Institute of Technology and Beth Israel Deaconess Medical Center.

### 2.2. Study population and group stratification

The inclusion criteria included: (1) age ≥ 65; (2) length of stay in ICU ≥ 24 h. Patients with missing key data (height, weight or albumin) on the first day of admission were excluded from the study. For patients with multiple hospitalizations, we only used their first hospitalization. The GNRI was calculated with the following formula (10): GNRI = 1.489 × serum albumin (g/L) + 41.7 × present weight (kg)/ideal weight (kg). The ideal body weight was calculated according to the Lorentz equations (10): 0.75 × height (cm) – 62.5 for men and 0.60 × height (cm) – 40 for women. When present weight exceeded ideal weight, present weight/ideal weight was set to 1. Patients were stratified into four groups according to the GNRI values, namely, major risk (GNRI: <82), moderate risk (GNRI: 82 to <92), low risk (GNRI: 92 to ≤98), and no risk (GNRI: >98) (10).

### 2.3. Outcome

The primary outcome was all-cause mortality in hospital. The secondary outcomes included ICU mortality, length of stay (LOS) in ICU and LOS in hospital.

### 2.4. Data extraction

The PostgreSQL 10.7 software and Structured Query Language were used to extract the baseline data within the first 24 h of ICU admission from the MIMIC-IV database. The following variables were collected, (1) demographic characteristics (age, gender, height, weight); (2) laboratory indicators (white blood cell (WBC) count, platelet count, hemoglobin, alanine transaminase (ALT), aspartate aminotransferase (AST) international normalized ratio (INR), serum creatinine (sCr), blood urea nitrogen (BUN), serum sodium, serum potassium, serum chloride, bicarbonate, anion gap and lactate); (3) comorbidities were identified according to International Classification of Diseases, 9th revised (ICD-9) and 10th revised (ICD-10) editions (chronic obstructive pulmonary disease (COPD), congestive heart failure (CHF), myocardial infarction (MI), chronic kidney disease (CKD), cirrhosis, cerebral infarction, malignancy and sepsis); (4) clinical severity scales (Sequential Organ Failure Assessment (SOFA) score and Simplified Acute Physiology Score II (SAPS II)); (5) treatment measures (renal replacement therapy (RRT) and mechanical ventilation (MV)).

### 2.5. Statistical analysis

Continuous variables are presented as the mean ± standard deviation (SD) for normal distribution and as the median and interquartile range (IQR) for skewed distribution. Normal distributions were confirmed by Shapiro–Wilk test. Continuous variables were compared by one-way ANOVA or Kruskal-Wallis H test, respectively. Categorical variables were compared using the χ^2^-test or Fisher exact test as appropriate.

Multivariable Cox proportional hazard models were used to examine the hazard ratios (HRs) and 95% confidence intervals (CIs) for associations between predefined GNRI groups and mortality. Model 1 was adjusted for age, gender and laboratory indicators. Model 2 was adjusted for variables in model 1 plus comorbidities and treatment measures. Model 3 was adjusted for variables in model 2 plus clinical severities. The assumption of the proportional hazards analysis was confirmed graphically by log cumulative hazard plots for mortality based on GNRI category. *p* for trend test was conducted by including the levels of GNRI as an ordinal score to the regression models. Restricted cubic spline (RCS) is a powerful tool to characterize a dose–response association between a continuous exposure and an outcome. RCS divide the observed range of the continuous variable with k knots and create a third order polynomial above the knot. RCS fit smoothly at each knot and to be linear both below the first knot and above the last knot. The knots are usually located at fixed percentiles of the continuous variable (17). So, the associations between continuous scale of GNRI and mortality were evaluated by RCS based on Cox proportional hazard models with three knots at the 10^th^, 50^th^ and 90^th^ percentiles of the distribution, adjusting for covariates in Model 3. The functional form of associations was evaluated by a Wald test comparing a linear or nonlinear likelihood ratio. Results were reported in log-relative hazard ratios and associated 95% CIs. If there were nonlinearity, we would further apply a two-piecewise linear regression model to examine the inflection point of GNRI on mortality, which provided maximum model likelihood. Finally, propensity score matching (PSM) was used to reduce selection bias in observational studies (18). Patients were matched in a 1:1 ratio with a caliper of 0.1 standard deviations of the Cox of the estimated propensity score with hospital mortality. Confounding factors such as age, gender, laboratory indicators, comorbidities, clinical severity scales and treatment measures were selected for matching.

Multivariable linear regression was used to analyze the relationship between GNRI (both as continuous and categorical variables) with LOS in hospital and ICU. Subgroup analyses according to gender, COPD, CHF, MI, CKD, cirrhosis, cerebral infarction, malignancy, sepsis (as defined by the Sepsis-3 criteria (19)), RRT and MV were conducted to test their interactions with GNRI on primary endpoint. GNRI was standardized to a Z-score ((GNRI- mean value)/SD) in order to present the confidence intervals of each subgroup clearly.

All data analyses were performed using R software (version 4.2.0; R Foundation for Statistical Computing, Vienna, Austria) and a two-sided *p*-value < 0.05 was considered statistically significant for all analyses. Variables with missing values were imputed using the multiple imputation method.

## 3. Results

### 3.1. Baseline characteristics

**Figure 1:**
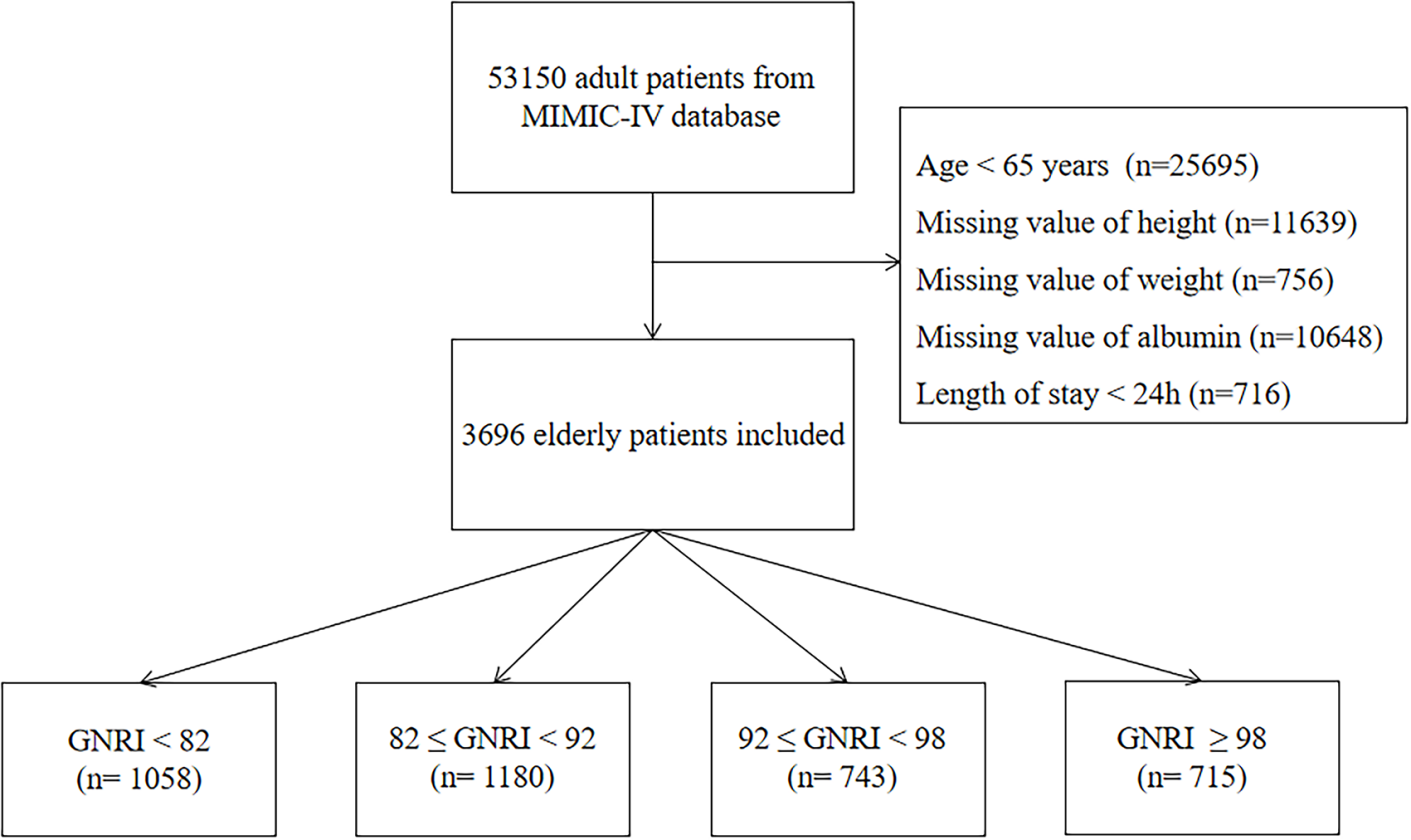
Flow chart of patients selection.

According to the inclusion criteria, a total of 3,696 elderly patients were finally obtained in the study (Figure 1). The median age of enrolled patients was 75 (IQR, 69–81) years with 2,095 (55.9%) male patients. Based on GNRI stratification, 1058 (28.6%) patients were in major risk group, 1180 (31.9%) patients were in moderate risk group, 743 (20.1%) patients were in mild risk group and 715 (19.3%) patients were in no risk group. The baseline characteristics of study population stratified by GNRI were shown in Table 1. With the decreasing of GNRI, patients with nutritional risk in ICU tended to be older and more likely to be female. In terms of laboratory indicators, patients in major risk group featured higher levels of WBC count, ALT, AST, bilirubin, INR, creatinine, BUN, and lactate and lower levels of hemoglobin, platelet count and bicarbonate compared with patients in no risk group. The prevalence of cirrhosis, sepsis and malignancy were more common in patients in major risk group. RRT and MV were used more frequently in patients with major nutritional risk. Clinical severities increased significantly with the decreasing of GNRI. When compared with patients in no risk group, patients in major risk group had significantly higher hospital mortality (30.7 vs. 15.2%, *p* < 0.001) and ICU mortality (24.3 vs. 11.7%, *p* < 0.001), and longer LOS in ICU (4.3, IQR (2.3–8.6) vs. 3.4, IQR (2.0–6.4), *p* < 0.001) and LOS in hospital [10.0, IQR (6.0–17.0) vs. 7.0, IQR (5.0–13.0), *p* < 0.001].

**Table 1:** Baseline characteristics of the study population grouped according to GNRI.

Characteristics	GNRI				*p*-value
	<82 (*n* = 1058)	82 to <92 (*n* = 1180)	92 to ≤98 (*n* = 743)	>98 (*n* = 715)	
Age	75.0 (69.0–81.0)	76.0 (70.0–81.0)	75.0 (70.0–81.0)	74.0 (69.0–80.0)	0.005
Male, *n* (%)	555 (52.5)	651 (55.2)	421 (56.7)	438 (61.3)	0.003^a^
*Laboratory indicators*					
WBC count (10^3^/μl)	10.5 (6.8–15.1)	9.8 (7.1–13.5)	8.80 (6.7–11.8)	8.7 (6.7–11.3)	<0.001
Hemoglobin (g/dl)	8.8 (7.5–10.1)	9.7 (8.4–11.2)	10.3 (8.9–11.8)	11.1 (9.6–12.9)	<0.001
Platelet count (10^3^/μl)	157.0 (99.0–238.0)	166.0 (115.0–232.2)	171.0 (127.00–227.5)	172.0 (134.0–220.0)	0.002
ALT (U/L)	33.0 (16.0–97.0)	28.0 (16.0–76.0)	23.0 (15.00–47.2)	22.0 (16.0–34.0)	<0.001
AST (U/L)	50.0 (28.0–146.00)	44.0 (26.0–121.5)	37.0 (23.0–76.0)	30.0 (22.0–52.0)	<0.001
Bilirubin (mg/dl)	0.8 (0.5–1.8)	0.7 (0.4–1.3)	0.6 (0.4–1.1)	0.6 (0.4–1.0)	<0.001
INR	1.5 (1.3–1.9)	1.3 (1.2–1.7)	1.3 (1.2–1.6)	1.2 (1.1–1.5)	<0.001
Creatinine (mg/dl)	1.4 (0.9–2.4)	1.4 (0.9–2.2)	1.2 (0.9–1.8)	1.1 (0.9–1.6)	<0.001
BUN (mg/dl)	34.0 (20.0–51.0)	29.0 (20.0–47.0)	24.0 (18.0–42.0)	23.0 (17.0–34.0)	<0.001
Bicarbonate (mmol/L)	19.0 (16.0–22.0)	21.0 (18.0–24.0)	21.0 (19.0–24.0)	22.0 (19.0–24.0)	<0.001
Anion gap (mmol/L)	17.0 (14.0–21.0)	17.0 (14.0–20.0)	16.0 (14.0–19.0)	17.0 (15.0–20.0)	0.357
Sodium (mmol/L)	140.0 (137.0–143.0)	140.0 (138.0–143.0)	140.0 (138.0–143.0)	140.0 (138.0–143.0)	0.207
Potassium (mmol/L)	4.6 (4.1–5.2)	4.5 (4.1–5.1)	4.5 (4.1–5.1)	4.5 (4.1–5.1)	0.310
Chloride (mmol/L)	103.0 (98.0–107.0)	102.0 (98.0–106.0)	102.0 (97.0–105.0)	101.0 (98.0–104.0)	<0.001
Lactate (mmol/L)	2.6 (1.6–5.4)	2.2 (1.4–4.0)	2.1 (1.3–3.5)	2.2 (1.6–3.6)	<0.001
*Comorbidities, n (%)*					
COPD	91 (8.6)	109 (9.2)	63 (8.5)	42 (5.9)	0.070^a^
CHF	318 (30.1)	472 (40.0)	301 (40.5)	213 (29.8)	<0.001^a^
MI	185 (17.5)	251 (21.3)	172 (23.1)	155 (21.7)	0.018^a^
CKD	193 (18.2)	242 (20.5)	140 (18.8)	110 (15.4)	0.049^a^
Cirrhosis	109 (10.3)	84 (7.1)	38 (5.1)	32 (4.5)	<0.001^a^
Cerebral infarction	43 (4.1)	44 (3.7)	45 (6.1)	51 (7.1)	0.002^a^
Malignancy	367 (34.7)	385 (32.6)	192 (25.8)	171 (23.9)	<0.001^a^
Sepsis	878 (83.0)	882 (74.7)	458 (61.6)	416 (58.2)	<0.001^a^
*Clinical severities*					
SOFA	5.0 (3.0–8.0)	4.0 (2.0–7.0)	3.0 (1.0–6.0)	2.0 (1.0–5.0)	<0.001
SAPS II	49.0 (40.0–59.0)	44.0 (36.0–52.0)	39.0 (32.0–48.0)	37.0 (30.0–45.0)	<0.001
*Treatment, n (%)*					
RRT	111 (10.5)	89 (7.5)	37 (5.0)	27 (3.8)	<0.001^a^
MV	708 (66.9)	713 (60.4)	395 (53.2)	376 (52.6)	<0.001^a^
*Outcomes*					
Hospital mortality, *n* (%)	325 (30.7)	259 (21.9)	130 (17.5)	109 (15.2)	<0.001^a^
ICU mortality, *n* (%)	257 (24.3)	199 (16.9)	109 (14.7)	84 (11.7)	<0.001^a^
LOS in hospital	10.0 (6.0–17.0)	9.0 (6.0–15.0)	7.0 (5.0–13.0)	7.0 (5.0–13.0)	<0.001
LOS in ICU	4.3 (2.3–8.6)	4.2 (2.1–7.7)	3.6 (2.0–6.3)	3.4 (2.0–6.4)	<0.001

Values were shown as median (interquartile range) unless otherwise indicated. ALT, alanine transaminase; AST, aspartate aminotransferase; BUN, blood urea nitrogen; CHF, congestive heart failure; CKD, chronic kidney disease; COPD, chronic obstructive pulmonary disease; GNRI, geriatric nutritional risk index; INR, international normalized ratio; LOS, length of stay; MI, myocardial infarction; MV, mechanical ventilation; RRT, renal replacement therapy; SAPSII, Simplified Acute Physiology Score II; SOFA, Sequential Organ Failure Assessment; WBC, white blood cell.

a *χ*^2^-test.

### 3.2. Multivariable Cox regression analyses between GNRI and all-cause mortality

As shown in model 1, after adjusting for age, gender and laboratory indicators, multivariable Cox proportional hazard models demonstrated significant negative associations between GNRI categories and hospital mortality (major risk vs. moderate risk [HR = 0.79, 95% CI: 0.65–0.96]; vs. mild risk [HR = 0.74, 95%: CI 0.58–0.96]; vs. no risk [HR = 0.66, 95% CI: 0.49–0.88]; *p* for trend 0.002). In model 2, after adjusting for variables in model 1 plus comorbidities and treatment measures, GNRI categories still had significantly negative associations with all-cause hospital mortality (major risk vs. moderate risk [HR = 0.79, 95% CI: 0.65–0.96]; vs. mild risk [HR = 0.75, 95% CI: 0.58–0.97]; vs. no risk [HR = 0.66, 95% CI: 0.50–0.89]; *p* for trend 0.026). However, in model 3, after adjusting for variables in model 2 plus clinical severities, GNRI categories only featured negative trend with hospital mortality (*p* for trend 0.037). Besides, there were no significant correlations between GNRI categories and ICU mortality in model 3 (Table 2).

**Table 2:** Cox proportional hazard models of the relationship between GNRI and all-cause mortality.

Categories	Model 1^*^	*p* value	*p* for trend	Model 2^†^	*p* value	*p* for trend	Model 3^‡^	*p* value	*p* for trend
	HR (95% CIs)			HR (95% CIs)			HR (95% CIs)		
**Hospital mortality**									
Major risk	1.00		0.002	1.00		0.002	1.00		0.037
Moderate risk	0.79 (0.65, 0.96)	0.016		0.79 (0.65, 0.96)	0.020		0.83 (0.68, 1.02)	0.070	
Low risk	0.74 (0.58, 0.96)	0.021		0.75 (0.58, 0.97)	0.026		0.81 (0.63, 1.05)	0.117	
No risk	0.66 (0.49, 0.88)	0.005		0.66 (0.50, 0.89)	0.006		0.75 (0.56, 1.01)	0.059	
**ICU mortality**									
Major risk	1.00		0.024	1.00		0.016	1.00		0.141
Moderate risk	0.80 (0.65, 0.98)	0.035		0.81 (0.65, 0.99)	0.046		0.85 (0.68, 1.07)	0.161	
Low risk	0.75 (0.57, 0.98)	0.040		0.75 (0.57, 0.99)	0.038		0.83 (0.62, 1.10)	0.191	
No risk	0.71 (0.52, 0.97)	0.025		0.71 (0.52, 0.98)	0.028		0.81 (0.59, 1.13)	0.214	

* Model 1: adjusted for age and gender and laboratory indicators (WBC, hemoglobin, platelet count, ALT, AST, bilirubin, INR, sCr, BUN, bicarbonate, anion gap, sodium, potassium, chloride, and lactate).

† Model 2: adjusted for model 1 plus comorbidities (COPD, CHF, MI, CKD, cirrhosis, cerebral infarction, malignancy and sepsis) and treatment measures (RRT and MV).

‡ Model 3: adjusted for model 2 plus clinical severities (SOFA and SAPS II).

### 3.3. Dose–response association between GNRI and all-cause mortality

**Figure 2:**
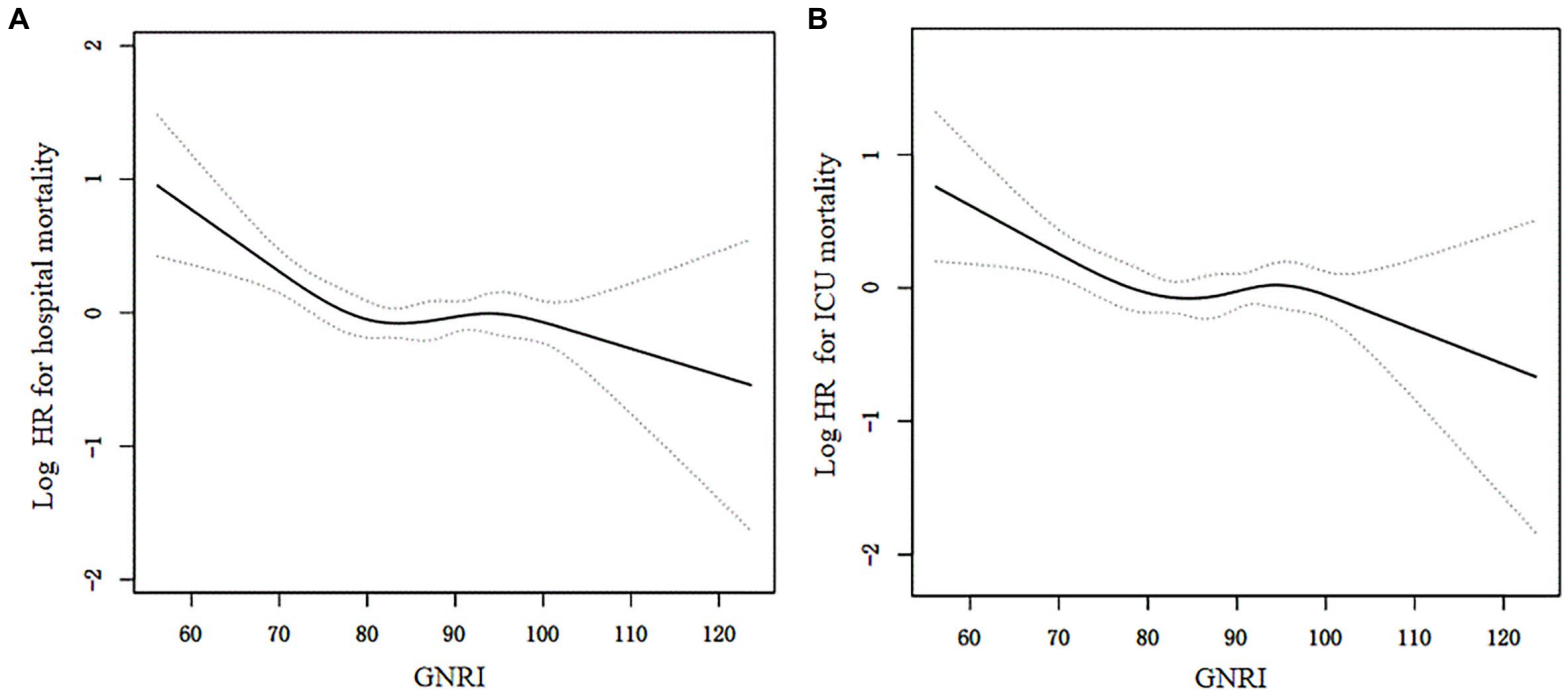
The associations of GNRI with hospital mortality **(A)** and ICU mortaltiy **(B)** by restricted cubic spline. The resulting figures showed the predicted log hazard ratios (HR) in the y-axis and the continuous levels of GNRI in the x-axis. The solid line represented the log hazard ratio and the dotted line was the 95% confidence interval (CI). HRs and associated 95% CIs were adjusted for variables in model 3.

On a continuous scale of GNRI, restricted cubic spline in a fully adjusted model showed that the associations of GNRI levels with all-cause hospital mortality (*p* for non-linearity = 0.003) and ICU mortality (*p* for non-linearity = 0.032) were L-shaped (Figure 2). The two-piecewise linear regression models indicated that the inflection points of GNRI for hospital and ICU mortality were 78.7 and 78.9, respectively. At the left side of inflection point, GNRI levels were significantly negatively associated with hospital mortality (HR = 0.96, 95% CI: 0.94–0.98; *p* < 0.001) and ICU mortality (HR = 0.97, 95% CI: 0.94–0.99; *p* = 0.006). While, at the right side of inflection point, GNRI levels had no significant relations with hospital or ICU mortality (Table 3).

**Table 3:** Threshold effect of GNRI on all-cause hospital and ICU mortality.

	Hospital mortality	*p* value	ICU mortality	*p* value
Inflection point	78.7		78.9	
< Inflection point HR (95% CI)	0.96 (0.94, 0.98)	<0.001	0.97 (0.94, 0.99)	0.007
≥Inflection point HR (95% CI)	1.00 (0.99, 1.01)	0.814	1.00 (0.99, 1.01)	0.848
*p* for log likelihood ratio test		0.012		0.048

Data were presented as hazard ratios (HR) and 95% confidence intervals (CI). The two-piecewise linear regression models were adjusted for age, gender, laboratory indicators (WBC, hemoglobin, platelet count, ALT, AST, bilirubin, INR, sCr, BUN, bicarbonate, anion gap, sodium, potassium, chloride, and lactate), comorbidities (COPD, CHF, MI, CKD, cirrhosis, cerebral infarction, malignancy and sepsis), treatment measures (RRT and MV) and clinical severities (SOFA and SAPS II).

After PSM, 778 patients in the non-survivor group were matched with 778 patients in the survivor group. The baseline profiles were well balanced between the two groups with standardized mean differences <10% for most of the variables (Additional file 1: Supplementary Table 1). Restricted cubic spline in a fully adjusted model also revealed an “L-shaped” relation (*p* for non-linearity = 0.004) between GNRI and hospital mortality (Additional file 1: Supplementary Figure 1).

### 3.4. Subgroup analyses of GNRI levels on hospital mortality

**Figure 3:**
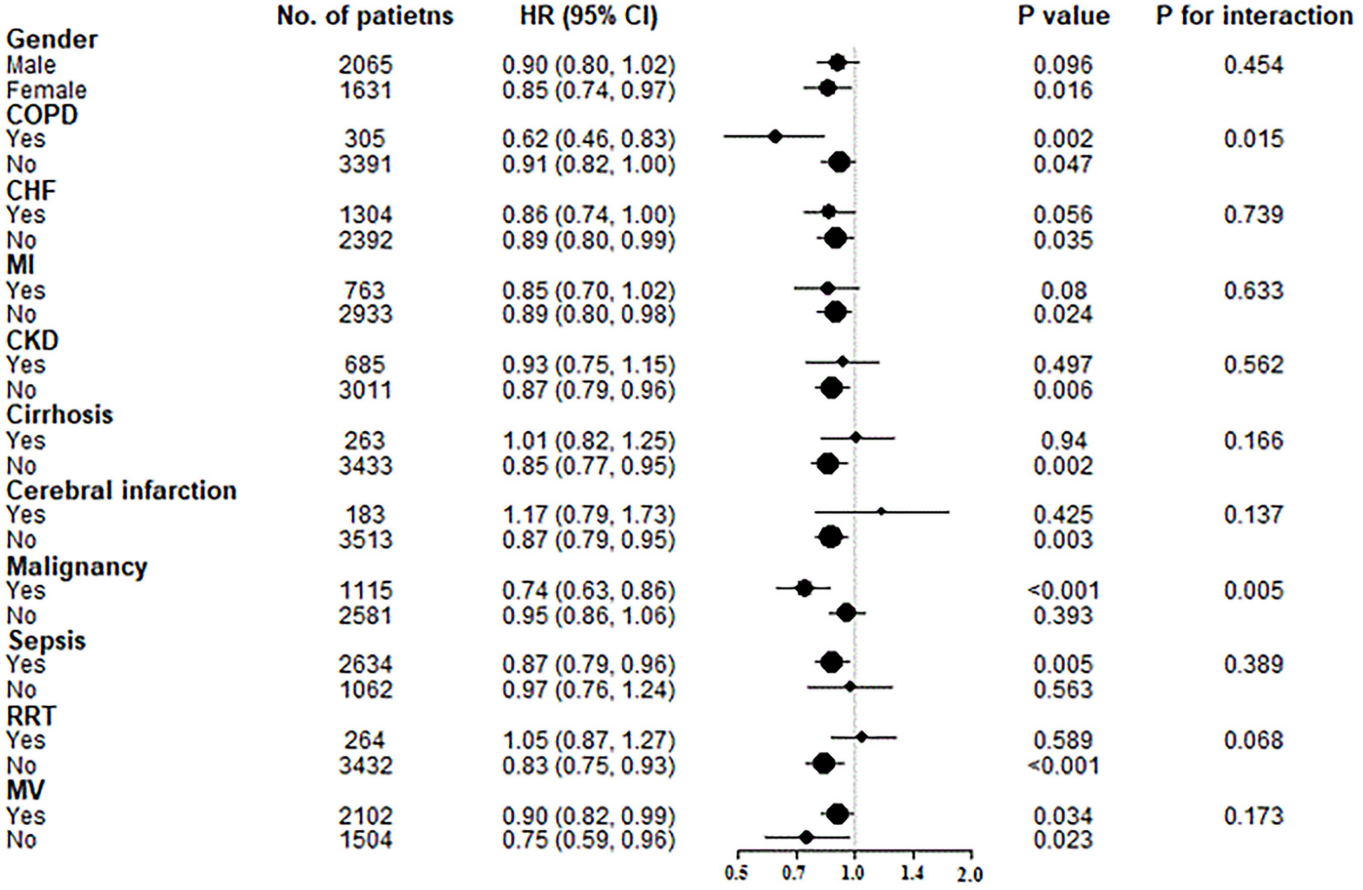
Subgroup analyses of the associations between GNRI levels and hospital mortality. GNRI was standardized to a *Z*-score. Above models were adjusted for variables in model 3. In each case, the model was not adjusted for stratification variable. COPD, chronic obstructive pulmonary disease; CHF, chronic heart failure; HI, myocardial infarction; CKD, chornic kidney disease; RRT, renal replacement therapy; MV, mechanical ventilation.

To further investigate possible interactions between GNRI levels and hospital mortality, several subgroup analyses were conducted according to gender, COPD, CHF, MI, CKD, cirrhosis, cerebral infarction, malignancy, sepsis, RRT and MV (Figure 3). After Z-transform standardization, significant interactions were observed in the subgroups of COPD (*p* for interaction =0.015) and malignancy (*p* for interaction =0.005). Elderly patients with COPD (HR = 0.62, 95% CI: 0.46–0.83; per unit increase in Z-score) and malignancy (HR = 0.74, 95% CI: 0.63–0.86; per unit increase in Z-score) featured stronger associations between GNRI levels and hospital mortality.

### 3.5. Relationship between GNRI and length of stay in hospital and ICU

As both category and continuous variables, multiple linear regression showed that GNRI was negatively associated with length of stay in hospital even after adjustment for age, gender and clinical severities among patients who were survival. However, after adjustment for age, gender and clinical severities, there was no significant relation between GNRI and length of stay in ICU (Table 4).

**Table 4:** Multivariable linear regression of the association between GNRI and length of stay.

	Length of stay in hospital^a^				Length of stay in ICU^b^			
	Crude model		Adjusted model^*^		Crude model		Adjusted model^*^	
	β (95% CI)	*p* value	β (95% CI)	*p* value	β (95% CI)	*p* value	β (95% CI)	*p* value
**GNRI categories**								
Major risk	1.00		1.00		1.00		1.00	
Moderate risk	−1.88 (−3.25, −0.51)	0.007	−0.73 (−2.08, −0.62)	0.291	−0.67 (−1.29, −0.04)	0.036	−0.07 (−0.68, 0.54)	0.823
Low risk	−4.06 (−5.58, −2.54)	<0.001	−1.94 (−3.47, −0.42)	0.012	−1.43 (−2.13, −0.74)	<0.001	−0.33 (−1.02, 0.37)	0.357
No risk	−3.96 (−5.49, −2.44)	<0.001	−1.60 (−3.15, −0.06)	0.042	−1.39 (−2.09, −0.69)	<0.001	−0.09 (−0.79, 0.62)	0.813
**GNRI continuous**								
GNRI levels	−0.18 (−0.23, −0.13)	<0.001	−0.09 (−0.14, −0.04)	<0.001	−0.06 (−0.09, −0.04)	<0.001	−0.01 (−0.04, 0.01)	0.253

* Adjusted for age, gender and clinical severities (SOFA and SAPS II).

a The association between GNRI and length of hospital stay was analyzed in patients who survived the hospital stay (*n* = 2873).

b The association between GNRI and length of ICU stay was analyzed in patients who survived the ICU stay (*n* = 3047).

## 4. Discussion

GNRI was transformed from nutritional risk index (NRI), which was introduced by Buzby et al. in 1988 to evaluate the severity of postoperative complications and malnutrition in hospitalized adults (20). NRI consists of serum albumin concentration and weight loss. However, it is difficult for elderly patients to recall their usual weight. Hence, Bouillanne et al. replaced usual body weight with ideal body weight using the Lorentz formula and developed a novel nutritional index, namely GNRI (10). GNRI is a “nutrition-related” risk index rather than an index of malnutrition. So, GNRI can be used to classify patients according to a risk of nutrition-related mortality, not as a tool for grading nutritional status (10). In recent years, due to the development of nutritional support theory, emerging studies have found that GNRI was a useful tool to screen for malnutrition-related mortality among geriatric patients in different complications (11–15). However, as a novel nutritional index, the investigation of GNRI focusing on critically ill patients is limited. In daily practice, it is important for clinicians to identify high-risk malnutrition patients who would be more likely to get benefit from nutritional support. However, preexistence of cognitive impairment at ICU admission ranges from 6 to 42% among older patients (21, 22). It is impossible for these patients to complete a series of questionnaires which are needed by several evaluation tools, such as Subjective Global Assessment (SGA) and MNA (23). Other screening tools depend on weight and dietary changes, which are often difficult to obtain in ICU. So, compared with NRS-2002, NUTRIC score, SGA and MNA, GNRI is clearly simple, less time-consuming and requires minimal participation by patients.

In this retrospective study with a total of 3,696 geriatric patients, we investigated the relationship between GNRI score (at admission to ICU) and hospital mortality. The median age of included patients was 75 (IQR, 69–81). The prevalence of major malnutrition risk assessed by GNRI was 28.6%. Compared with patients in no risk group, patients in major risk groups had significantly higher ICU mortality, hospital mortality and longer duration of stay in ICU and hospital. This result was further supported by restricted cubic spline curves and we found an L-shaped association between continuous GNRI levels and the risk of all-cause mortality. Previous studies only investigated prognostic value of GNRI by focusing on specific ICU population, such as acute respiratory failure (9), stroke (24), and trauma (25). Therefore, the optimal cutoff value of GNRI suitable for general elderly patients remains to be elucidated. With the aid of two-piecewise linear regression models, we found that GNRI was significantly negatively associated with hospital mortality when it was less than 78.7 (HR = 0.96, 95% CI: 0.94–0.98) in the fully adjusted model. As mentioned with previous studies (26, 27), our study also found that GNRI had the ability to predict LOS in hospital. Therefore, elderly patients with malnutrition risk at admission to ICU tended to have a longer duration of stay in hospital.

Then, we further conducted subgroup analyses to find interaction effect and observed that GNRI featured a stronger relation with hospital mortality in patients with COPD or malignancy. For geriatric COPD patients, GNRI may be useful to be applied as a nutritional assessment scale (28, 29). As regard to malignancy, two meta-analyses concluded that low GNRI level was correlated with poor overall survival in patients with gastrointestinal malignancy (30) and lung cancer (31). Other studies also found its prognostic value in hepatocellular carcinoma (32), renal cancer (33), bladder cancer (34), and lymphoma (35, 36). Similarly, these results indicated clinical value of GNRI in nutrition assessment among elderly cancer patients.

Several limitations of this study should be considered. First, it was a single-center retrospective study. Prospective studies by multi-center are needed to validate the generalizability of the findings in the future. Second, the data were extracted from electronic database, missing important information in a certain of patients is evitable. Third, some useful indicators are incomplete, such as C-reactive protein (CRP), procalcitonin and B-type natriuretic peptide. So, these confounders were not adjusted in our model. Fourth, modified GNRI was developed recently by using the inverse of CRP instead of albumin (37). Due to the insufficient data of CRP, we were not able to make comparison of prognostic value between GNRI and modified GNRI. Last but not least, we did not make comparisons of diagnostic value among GNRI, NRS-2002, NUTRIC score, MNA and SGA. Further study needs to investigate which screening tool could provide more significant prognostic value in the critical care setting for elderly patients.

## 5. Conclusion

This study demonstrated that the associations of GNRI levels with hospital and ICU mortality were L-shaped. GNRI levels were negatively correlated with hospital and ICU mortality when its value was less than 79, which was slightly lower than that used for major risk. As a simple screening tool for malnutrition risk, the major risk of malnutrition defined by GNRI was able to predict poor prognosis for geriatric patients admitted to ICU, which allowed clinicians to identify suitable patients for nutritional support.

## Data availability statement

The raw data supporting the conclusions of this article will be made available by the authors, without undue reservation.

## Ethics statement

The studies involving human participants were reviewed and approved by Institutional Review Boards of the Massachusetts Institute of Technology and Beth Israel Deaconess Medical Center. Written informed consent for participation was not required for this study in accordance with the national legislation and the institutional requirements.

## Author contributions

J-CP: writing—original draft preparation. Y-WZ: methodology. S-PX: validation. WL: formal analysis. YG: investigation. W-WG: writing—review and editing. All authors contributed to the article and approved the submitted version.

## Conflict of interest

The authors declare that the research was conducted in the absence of any commercial or financial relationships that could be construed as a potential conflict of interest.

## Publisher’s note

All claims expressed in this article are solely those of the authors and do not necessarily represent those of their affiliated organizations, or those of the publisher, the editors and the reviewers. Any product that may be evaluated in this article, or claim that may be made by its manufacturer, is not guaranteed or endorsed by the publisher.

## Figures and Tables

**Table:** 

ALT	Alanine transaminase
APACHE	Acute physiology and chronic health evaluation II
ASPEN	American society for parenteral and enteral nutrition
AST	Aspartate aminotransferase
BMI	Body mass index
BUN	Blood urea nitrogen
CHF	Congestive heart failure
CI	Confidence interval
CKD	Chronic kidney disease
COPD	Chronic obstructive pulmonary disease
ESPEN	European society for clinical nutrition and metabolism
GNRI	Geriatric nutritional risk index
HR	Hazard ratio
ICD	International classification of diseases
ICU	Intensive care unit
INR	International normalized ratio
IQR	Interquartile range
LOS	Length of stay
MI	Myocardial infarction
MIMIC-IV	Medical information mart for intensive care IV
MNA	Mini nutritional assessment
MV	Mechanical ventilation
NRS-2002	Nutritional risk screening-2002
NUTRIC	Nutrition risk in the critically ill
RRT	Renal replacement therapy
sCr	Serum creatinine
SAPS II	Simplified acute physiology score II
SGA	Subjective global assessment
SD	Standard deviation
SOFA	Sequential organ failure assessment
WBC	White blood cell
