# Supplementary material for: Association of geriatric nutritional risk index with all-cause hospital mortality among elderly patients in intensive care unit
Source: Front Nutr. 2023 Mar 23;10:1117054. doi: 10.3389/fnut.2023.1117054 (PMC10076778; doi:10.3389/fnut.2023.1117054)
Supplement: Supplementary file 1 [file Data_Sheet_1.docx]

Table S1. Characteristics of participants before and after matching by hospital mortality.

| Characteristics | Pre-matched cohort | | *P*-value | Post-matched cohort | | SMD |
| --- | --- | --- | --- | --- | --- | --- |
|  | Survivors (n=2873) | Non-survivors (n=823) |  | Survivors (n=778) | Non-survivors (n=778) |  |
| Age | 75.0 (69.0-81.0) | 76.0 (70.0-82.0) | 0.002 | 78.0 (72.0-83.0) | 76.0 (70.0-82.0) | 0.235 |
| Male, n (%) | 1628 (56.7) | 437 (53.1) | 0.069 | 358 (46.0) | 412 (53.0) | 0.139 |
| Laboratory indicators | | | | | | |
| WBC count (10^3^/μL) | 9.2 (6.7-12.6) | 10.8 (7.5-14.9) | <0.001 | 11.1 (7.9-15.6) | 10.6 (7.5-14.8) | 0.084 |
| Hemoglobin (g/dL) | 9.9 (8.4-11.4) | 9.7 (8.1-11.2) | 0.024 | 9.8 (8.2-11.4) | 9.7 (8.2-11.2) | 0.051 |
| Platelet count (10^3^/μL) | 169.0 (119.0-230.0) | 157.0 (105.0-226.0) | <0.001 | 157.0 (109.0-220.0) | 159.0 (107.0-227.0) | 0.002 |
| ALT (U/L) | 24.0 (15.0-53.0) | 33.0 (17.0-117.5) | <0.001 | 33.0 (19.00-100.8) | 32.0 (17.0-96.6) | 0.022 |
| AST (U/L) | 37.0 (23.0-82.00) | 59.0 (31.0-199.0) | <0.001 | 55.0 (30.0-152.8) | 55.0 (30.0-160.8) | 0.026 |
| Bilirubin (mg/dL) | 0.7 (0.4-1.3) | 0.9 (0.5-1.7) | <0.001 | 0.8 (0.5-1.6) | 0.8 (0.5-1.6) | 0.010 |
| INR | 1.3 (1.2-1.6) | 1.5 (1.2-2.2) | <0.001 | 1.4 (1.2-2.0) | 1.5 (1.2-2.1) | 0.043 |
| Creatinine (mg/dL) | 1.2 (0.9-1.9) | 1.6 (1.1-2.7) | <0.001 | 1.5 (1.0-2.4) | 1.6 (1.1-2.6) | 0.042 |
| BUN (mg/dL) | 26.0 (18.0-41.0) | 38.0 (23.0-56.0) | <0.001 | 34.0 (23.0-55.0) | 37.0 (23.0-54.0) | 0.004 |
| Bicarbonate (mmol/L) | 21.0 (18.0-24.0) | 19.0 (15.0-22.0) | <0.001 | 20.0 (16.0-23.0) | 19.0 (16.0-23.0) | 0.024 |
| Anion gap (mmol/L) | 16.0 (14.0-19.0) | 19.0 (15.0-23.0) | <0.001 | 19.0 (16.0-22.0) | 19.0 (15.0-22.0) | 0.019 |
| Sodium (mmol/L) | 140.0 (138.0-143.0) | 140.0 (137.0-144.0) | 0.019 | 140.0 (137.0-143.0) | 140.0 (137.0-144.0) | 0.015 |
| Potassium (mmol/L) | 4.5 (4.1-5.2) | 4.5 (4.1-5.1) | <0.001 | 4.8 (4.3-5.4) | 4.7 (4.2-5.3) | 0.086 |
| Chloride (mmol/L) | 102.0 (98.0-106.0) | 101.0 (96.0-106.0) | <0.001 | 101.0 (97.0-105.0) | 101.0 (97.0-106.0) | 0.060 |
| Lactate (mmol/L) | 2.2 (1.4-3.7) | 2.8 (1.7-5.3) | <0.001 | 2.8 (1.7-5.0) | 2.7 (1.7-4.9) | 0.011 |
| Comorbidities, n (%) | | | | | | |
| COPD | 234 (8.1) | 71 (8.6) | 0.658 | 67 (8.6) | 68 (8.7) | 0.005 |
| CHF | 993 (34.6) | 311 (37.8) | 0.088 | 321 (41.3) | 296 (38.0) | 0.066 |
| MI | 576 (20.0) | 187 (22.7) | 0.095 | 206 (26.5) | 175 (22.5) | 0.093 |
| CKD | 532 (18.5) | 153 (18.6) | 0.962 | 154 (19.8) | 145 (18.6) | 0.029 |
| Cirrhosis | 172 (6.0) | 91 (11.1) | <0.001 | 68 (8.7) | 77 (9.9) | 0.040 |
| Cerebral infarction | 126 (4.4) | 57 (6.9) | 0.003 | 59 (7.6) | 56 (7.2) | 0.015 |
| Malignancy | 858 (29.9) | 257 (31.2) | 0.453 | 262 (33.7) | 239 (30.7) | 0.063 |
| Sepsis | 1937 (67.4) | 697 (84.7) | <0.001 | 615 (79.0) | 663 (85.2) | 0.162 |
| Clinical severities | | | | | | |
| SOFA | 4.0 (2.0-6.0) | 6.0 (3.0-9.0) | <0.001 | 6.0 (3.0-9.0) | 6.0 (3.0-8.0) | 0.012 |
| SAPS II | 40.0 (33.0-49.0) | 53.0 (43.0-63.0) | <0.001 | 53.0 (46.0-62.0) | 52.0 (42.0-61.0) | 0.039 |
| Treatment, n (%) | | | | | | |
| RRT | 109 (3.8) | 155 (18.8) | <0.001 | 83 (10.7) | 133 (17.1) | 0.187 |
| MV | 1523 (53.0) | 669 (81.3) | <0.001 | 652 (83.8) | 626 (80.5) | 0.087 |
| GNRI categories, n (%) <0.001 | | | | | | |
| Major risk  (GNRI< 82) | 733 (25.5) | 325 (39.5) |  | 254 (32.6) | 302 (38.8) | 0.129 |
| Moderate risk  (GNRI 82 to < 92) | 921 (32.1) | 259 (31.5) |  | 269 (34.6) | 245 (31.5) | 0.066 |
| Low risk  (GNRI 92 to ≤ 98), | 613 (21.3) | 130 (15.8) |  | 136 (17.5) | 124 (15.9) | 0.041 |
| No risk  (GNRI > 98) | 606 (21.1) | 109 (13.2) |  | 119 (15.3) | 107 (13.8) | 0.044 |

*Values were shown as median (interquartile range) unless otherwise indicated.*

*ALT, alanine transaminase; AST, aspartate aminotransferase; BUN, blood urea nitrogen; CHF, congestive heart failure; CKD, chronic kidney disease; COPD, chronic obstructive pulmonary disease; GNRI, geriatric nutritional risk index; INR, international normalized ratio; LOS, length of stay; MI, myocardial infarction; MV, mechanical ventilation; RRT, renal replacement therapy; SAPSII, Simplified Acute Physiology Score II; SMD, standardized mean difference; SOFA, Sequential Organ Failure Assessment; WBC, white blood cell.*


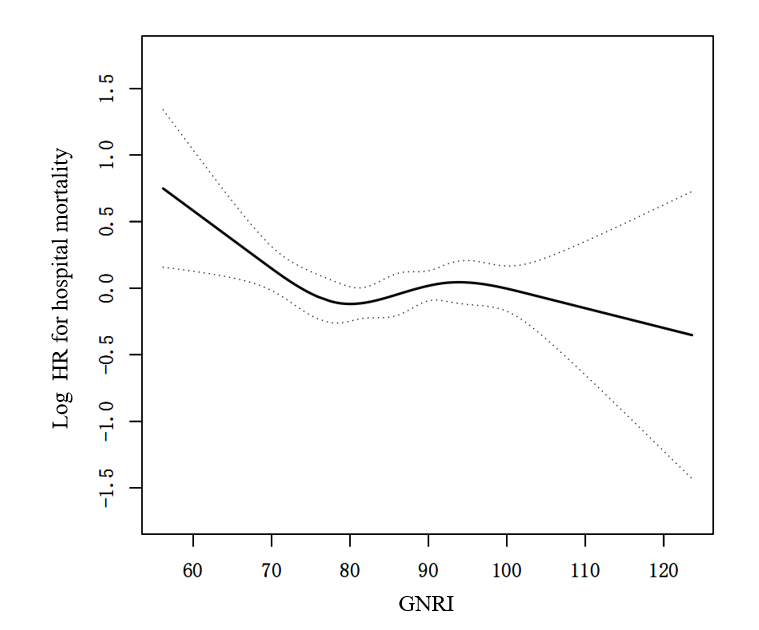


Figure S1. The associations of GNRI with hospital mortality by restricted cubic spline after propensity-score matching. The resulting figures showed the predicted log hazard ratios (HR) in the y-axis and the continous GNRI in the x-axis. HRs and associated 95% CIs were adjusted for age, gender, laboratory indicators, comorbidities, treatment measures and clinical severities.
